# Supplementary material for: Nanotwin architecture and ultra-high valley degeneracy lead to high thermoelectric performance in GeTe-based thermoelectric materials
Source: Nat Commun. 2026 Jan 31;17:2205. doi: 10.1038/s41467-026-68908-0 (PMC12963521; doi:10.1038/s41467-026-68908-0)
Supplement: Supplementary file 1 — Supporting Information [file 41467_2026_68908_MOESM1_ESM.pdf]

Supporting Information

Nanotwin Architecture and Ultra-high Valley Degeneracy Lead to High Thermoelectric Performance in GeTe-based Thermoelectric Materials

Song Li[1,#], Yuxuan Yang[2,#], Xiaoyu Fei[3,4], Yang Geng[1], Jiajun Nan[1], Pubao Peng[1], Guizhong Li[1], Yang Zhang[2,5], Xiaobing Liu[3,4], Yongsheng Zhang[3,4,*], Haijun Wu[2,*], Guodong Tang[1,*]

[1]School of Materials Science and Engineering, Nanjing University of Science and Technology, Nanjing, 210094, China.

[2]State Key Laboratory for Mechanical Behavior of Materials, Xi'an Jiaotong University, Xi'an, 710049, China.

[3]Key Laboratory of Quantum Materials under Extreme Conditions in Shandong Province, School of Physics and Physical Engineering, Qufu Normal University, Qufu 273165, China

[4]Laboratory of High Pressure Physics and Material Science (HPPMS), Advanced Research Institute of Multidisciplinary Sciences, Qufu Normal University, Qufu 273165, China

[5]Electronic Materials Research Laboratory (Key Lab of Education Ministry), School of Electronic and Information Engineering and Instrumental Analysis Center, Xi'an Jiaotong University, Xi'an, 710049, China

[#]These authors contributed equally: Song Li, Yuxuan Yang

*To whom correspond should be addressed.

e-mail: yshzhang@qfnu.edu.cn (Y. S. Zhang); wuhaijunnavy@xjtu.edu.cn (H. J. Wu); tangguodong@njust.edu.cn (G. D. Tang)

Supplementary Methods

The single parabolic (SPB) model in detail:

The Lorentz number L and effective mass m^* were estimated based on the SPB model and given by the following relationship:

The Lorenz number can be given as[1]:

$$L = \left(\frac{k_B}{e}\right)\left(\frac{(r+7/2)F_{r+5/2}(\eta)}{(r+3/2)F_{r+1/2}(\eta)} - \left[\frac{(r+5/2)F_{r+3/2}(\eta)}{(r+3/2)F_{r+1/2}(\eta)}\right]^2\right) \tag{1}$$

For the Lorenz number calculation, firstly, we should get reduced Fermi energy η. The calculation of η can be derived from the measured Seebeck coefficients by using the following relationship:

$$S = \pm\frac{k_B}{e}\left(\frac{(r+5/2)F_{r+3/2}(\eta)}{(r+3/2)F_{r+1/2}(\eta)} - \eta\right) \tag{2}$$

where $F_n(\eta)$ is the n-th order Fermi integral,

$$F_n(\eta) = \int_0^\infty \frac{\chi^n}{1+e^{\chi-\eta}} d\chi \tag{3}$$

effective mass:

$$m^* = \frac{h^2}{2k_B T}\left(\frac{n}{4\pi F_{1/2}(\mu)}\right)^{\frac{2}{3}} \tag{4}$$

where e is the electron charge, k_B is the Boltzmann constant, h is the Planck constant, and r is the scattering factor. Here, r is -1/2 since acoustic phonon scattering has been assumed as the main carrier scattering mechanism near room temperature (RT). Lorentz number can be obtained by combining equations (1), (2) and (3). Effective mass is calculated by (4).

Debye-Callaway Model in detail:

According to the Debye-Callaway model, κ_L can be calculated by[2]:

$$\kappa_L = \int_0^{\theta_D/T} \kappa_s\,(x)dx \tag{5}$$

$\kappa_s(x)$ denotes the spectral lattice thermal conductivity, which indicates the extent to which various scattering mechanisms affect phonons of different frequencies. The dimensionless parameter x is defined as: $x=\hbar\omega/(kBT)$ where: $\hbar$: Reduced Planck constant, ω: Phonon frequency ΘD: Debye temperature.

$$\kappa_s(\mathrm{x}) = \frac{k_B}{2\pi^2 v_{\mathrm{ave}}} \cdot \left(\frac{k_B T}{\hbar}\right)^3 \cdot \tau_x \frac{x^4\,exp(x)}{[exp(x)-1]^2} \tag{6}$$

The quantity $\tau(x)$ denotes the total relaxation time, which is collectively governed by various scattering mechanisms present within the thermoelectric material. According to Matthiessen's rule (MR), the total relaxation time equals the *sum* (in the reciprocal sense) of contributions from all individual scattering mechanisms:

$$\tau_{\mathrm{tot}}^{-1} = \tau_U^{-1} + \tau_N^{-1} + \tau_{PD}^{-1} + \tau_{GB}^{-1} + \tau_{TB}^{-1} + \tau_{VA}^{-1} + \cdots \tag{7}$$

where $\tau_U{}^{-1}$, $\tau_N{}^{-1}$, $\tau_{PD}{}^{-1}$, $\tau_{GB}{}^{-1}$ $\tau_{TB}{}^{-1}$ and $\tau_{VA}{}^{-1}$ are the contributions from the Umklapp phonon-phonon scattering, normal phonon-phonon scattering, point-defect scattering, grain boundary scattering, twin boundary scattering and vacancy array scattering respectively.

$$\tau_U^{-1} = \frac{\hbar\gamma^2}{M v_{\mathrm{ave}}\theta_D}\omega^2 T \cdot exp\left(-\frac{\theta_D}{3T}\right) \tag{8}$$

Here, γ and M represent the Grüneisen parameter and the mean atomic mass of the crystalline solid, respectively,

$$\tau_N^{-1} = \beta \cdot \tau_U^{-1} \tag{9}$$

β denotes the proportional constant characterizing the ratio between Umklapp scattering and Normal scattering processes,

$$\tau_{PD}^{-1} = \frac{\omega^4 \overline{V}}{4\pi v_{\mathrm{ave}}^3} \Gamma \tag{10}$$

the parameter Γ describes the mass and atomic size contrast with the lattice and $\overline{V}$ is the is the average atomic volume. The parameter Γ ($\Gamma = \Gamma_{\mathrm{M}} + \Gamma_{\mathrm{S}}$) describes the mass and atomic size contrast with the lattice and represents the strength of point defect phonon scatterings, which includes two components, the scattering parameters due to mass fluctuations Γ_{M} and strain field fluctuations Γ_{S}.[3]

$$\Gamma_M = x(1-x)\left(\frac{M_i - \bar{M}}{\bar{M}}\right)^2 \tag{11}$$

$$\Gamma_S = x(1-x)\varepsilon\left(\frac{a_i - \bar{a}}{\bar{a}}\right)^2 \tag{12}$$

$\bar{M}$ is the average atomic mass, $\bar{a}$ is the average atomic radius, ε is the phenomenological parameter. Grain boundary scattering:

$$\tau_{GB}^{-1} = \frac{v_{ave}}{d} \tag{13}$$

d is average grain size. Twin boundary scattering:

$$\tau_{TB}^{-1} = \frac{v_{ave}}{l} \tag{14}$$

l is average twin structure size. The average grain size (l) in the GeTe matrix after SPS sintering was determined to be approximately ~5 μm by statistical analysis of the polished surface using scanning electron microscopy (SEM). The average twin structure (d) size is estimated by transmission electron microscope (TEM) analysis to be ~90 nm. The uncertainty is dominated by the statistical distribution of sizes. We estimate 5% uncertainty in l and 5% uncertainty in d due to the limited sampling area in SEM and TEM. We incorporate the two scattering mechanisms together as: $\tau_B^{-1} =$

$\tau_{GB}^{-1} + \tau_{TB}^{-1}$.

We consider vacancy array as stacking fault to calculate relaxation time:

$$\tau_{VA}{}^{-1} = 0.7\frac{a_{lat}{}^2\gamma^2 N_{va}}{v_s}\omega^2 \tag{15}$$

where N_{va} is the density of ordered vacant seats, and a_{lat} is the average lattice parameter, The defect density N_{va} was estimated through TEM analysis by counting the number of vacancy arrays intersecting per unit length as ~ 4×10^6 m^{-1}. The main uncertainty arises from limited sampling area in TEM. We estimate 10% relative error in this measurement.

Weighted mobility calculation in detail:

Weighted mobility (μ_W) can be calculated based on the availability of combined electrical conductivity and Seebeck coefficient according to the following equation:

$$\mu_W = \frac{3h^3\sigma}{8\pi e(2m_e k_B T)^{3/2}}\left[\frac{exp\left[\frac{|S|}{k_B/e}-2\right]}{1+exp\left[-5\frac{|S|}{k_B/e}-1\right]} + \frac{\frac{3}{\pi^2}\left[\frac{|S|}{k_B/e}\right]}{1+exp\left[5\frac{|S|}{k_B/e}-1\right]}\right] \tag{16}$$

where T is the absolute temperature, k_B is the Boltzmann constant, e is the electron charge, h is the Planck constant, and m_e is the electron mass, respectively.

Supplementary Table 1. Rietveld refinement details of the $(GeTe)_{0.93}(CuBiS_2)_{0.07}$ sample.

Atom	Wyckoff position			Occupancy
	x	y	z	
Ge	0.0000	0.0000	0.2437	0.8856
Cu				0.0596
Bi				0.0548
Te	0.0000	0.0000	0.7629	0.8819
S				0.1181

Supplementary Table 2. Quantitative phase fractions extracted from the XRD Rietveld refinement for $(GeTe)_{1-x}(CuBiS_2)_x$ samples.

Composition	Phase fraction (%)		
	GeTe	Ge	$CuBiS_2$
GeTe	100	/	/
$(GeTe)_{0.97}(CuBiS_2)_{0.03}$	100	/	/
$(GeTe)_{0.95}(CuBiS_2)_{0.05}$	100	/	/
$(GeTe)_{0.93}(CuBiS_2)_{0.07}$	99.73	0.27	/
$(GeTe)_{0.91}(CuBiS_2)_{0.09}$	97.85	0.46	1.69

Supplementary Table 3. The theoretical densities calculation by XRD refinement and measured actual densities for $(GeTe)_{1-x}(CuBiS_2)_x$ samples.

Compositions	Actual Density (ρ, g/cm^3)	Theoretical Density (ρ, g/cm^3)	Density %
GeTe	5.9010	6.2109	95.01
$(GeTe)_{0.97}(CuBiS_2)_{0.03}$	5.9863	6.2080	96.43
$(GeTe)_{0.95}(CuBiS_2)_{0.05}$	6.0314	6.1934	97.38
$(GeTe)_{0.93}(CuBiS_2)_{0.07}$	6.0485	6.1788	97.89
$(GeTe)_{0.91}(CuBiS_2)_{0.09}$	6.0412	6.1691	97.93

Supplementary Table 4. Parameters for the lattice thermal conductivity of $(GeTe)_{0.93}(Cu_2BiS_2)_{0.07}$ sample in the Debye-Callaway model.

Parameters	Descriptions	Values
M_i	Atomic mass of matrix	72.63 g mol-1 (Ge)<br>127.6 g mol-1 (Te)
M	Atomic mass of impurities	63.55 g mol^{-1} (Cu)<br>208.98 g mol^{-1} (Bi)<br>32.07 g mol^{-1} (S)
v	Average speed of sound	2075 ms^{-1}
v_L	Longitudinal sound velocity	3266 ms^{-1}
v_T	Transverse sound velocity	1887 ms^{-1}
V_{cell}	Unit cell volume	161.82 Å^3
N_{cell}	Number of atoms in unit cell	6
β	Temperature-dependent ratio of normal phonon scattering to Umklapp scattering	1.5[4]
θ_D	Debye temperature	206 K
γ	Grüneisen parameter	2.19[5]
Gamma	Disorder scattering factor (dimensionless)	0.14
υ	Poisson's ratio	0.25
N_{va}	Number of vacant arrays a line of unit length	4×10^6 m^{-1}
d	Average grain size	5 µm
l	Average twin structure size	90 nm

Supplementary Table 5. The carrier concentration and Seebeck coefficient of $(GeTe)_{1-x}(CuBiS_2)_x$ samples at room temperature, alongside the density-of-states (DOS) effective mass deduced via single parabolic band (SPB) model fitting.

Compositions	Carrier Concentration (10^{20} cm^{-3})	Seebeck Coefficient ($\mu V\ K^{-1}$)	DOS effective mass (m_0)
GeTe	6.5	30.02	1.03
$(GeTe)_{0.97}(CuBiS_2)_{0.03}$	5.90	56.27	1.80
$(GeTe)_{0.95}(CuBiS_2)_{0.05}$	4.31	73.66	1.92
$(GeTe)_{0.93}(CuBiS_2)_{0.07}$	2.28	121.30	2.23

Supplementary Table 6. Reported valley degeneracy (N_v) realized in typical GeTe-based thermoelectric materials of *c*-GeTe structure.

Material	Contribution	Total degeneracy
c-GeTe[6]	N_v^{VBM1} =4 N_v^{VBM2} <12	< 16
$Ge_{0.85}Ca_{0.05}Sb_{0.1}Te$[7]	N_v^{VBM1} =4 N_v^{VBM2} <12	< 16
$Ge_{0.88}Cd_{0.05}Bi_{0.07}Te$[4]	N_v^{VBM1} =4 N_v^{VBM2} <12	< 16
$Ge_{0.86}Mn_{0.10}Sb_{0.04}Te$[8]	N_v^{VBM1} =4 N_v^{VBM2} <12	< 16
$Ge_{0.86}Sb_{0.1}Zn_{0.04}Te$[9]	N_v^{VBM1} =4 N_v^{VBM2} =12	16
$Ge_{0.9}V_{0.02}Bi_{0.08}Te$[10]	N_v^{VBM1} =4 N_v^{VBM2} =12	16
This work	N_v^{VBM1} =4 N_v^{VBM2} =12 N_v^{VBM3} =6	22

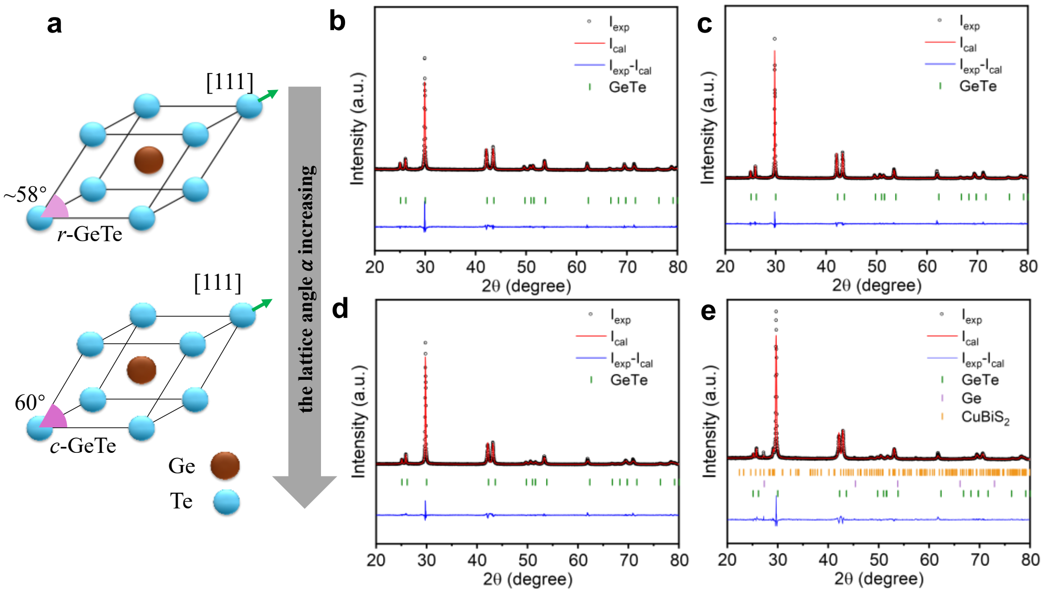

Supplementary Fig. 1. The crystal structure and Rietveld refinement. (a) Crystal structures of rhombohedral and cubic GeTe, Rietveld refinement details of the XRD pattern of (b) pure GeTe, (c) $(GeTe)_{0.97}(CuBiS_2)_{0.03}$, (d) $(GeTe)_{0.95}(CuBiS_2)_{0.05}$, (e) $(GeTe)_{0.91}(CuBiS_2)_{0.09}$.

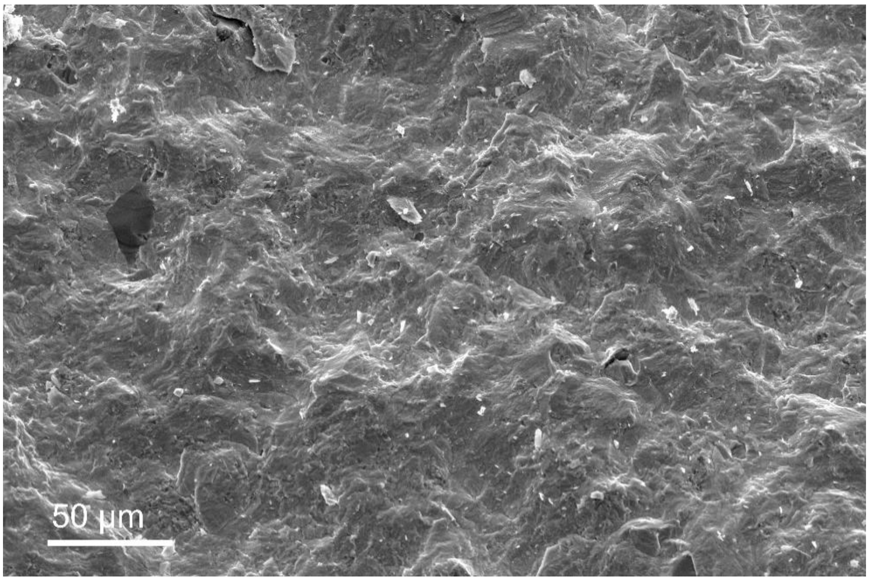

Supplementary Fig. 2. Scanning electron microscope (SEM) images of the fracture surface for $(GeTe)_{0.93}(CuBiS_2)_{0.07}$ sample.

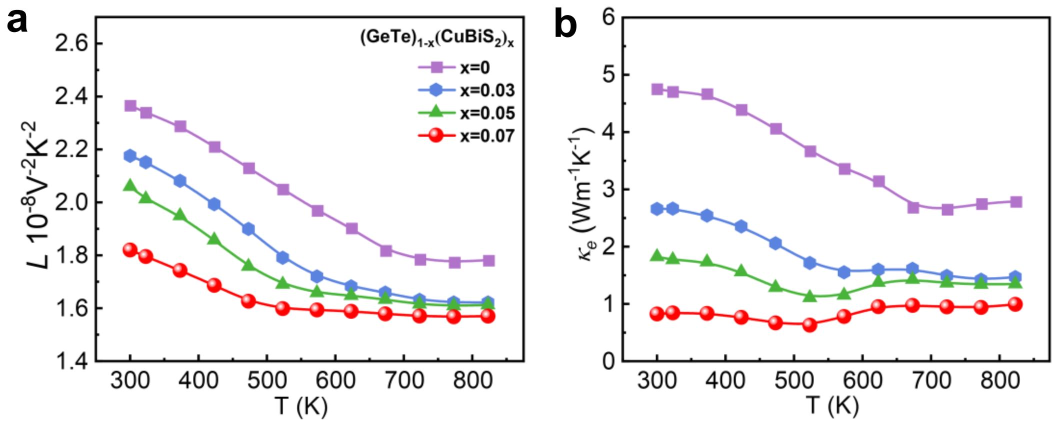

Supplementary Fig. 3. Thermal transport properties as a function of temperature for $(GeTe)_{1-x}(CuBiS_2)_x$ samples. (a) Lorenz number (L). (b) Electrical thermal conductivity (κ_e).

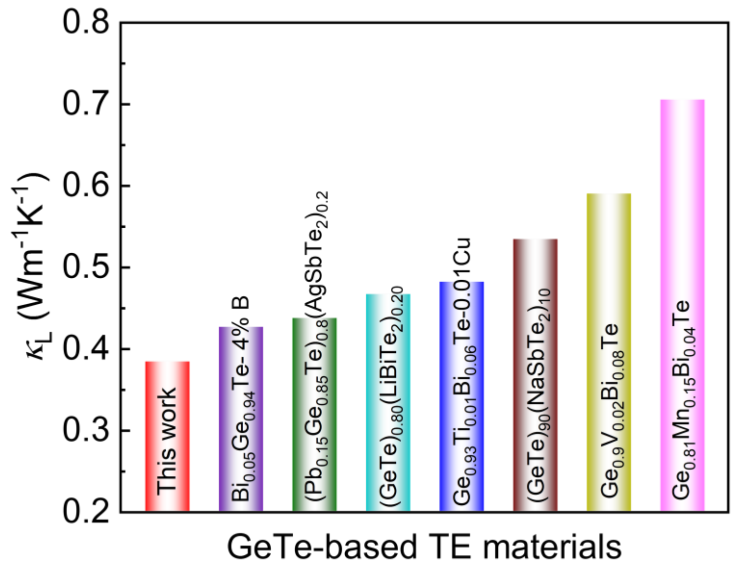

Supplementary Fig. 4. Comparison of the minimum κ_L between this work and representative works.

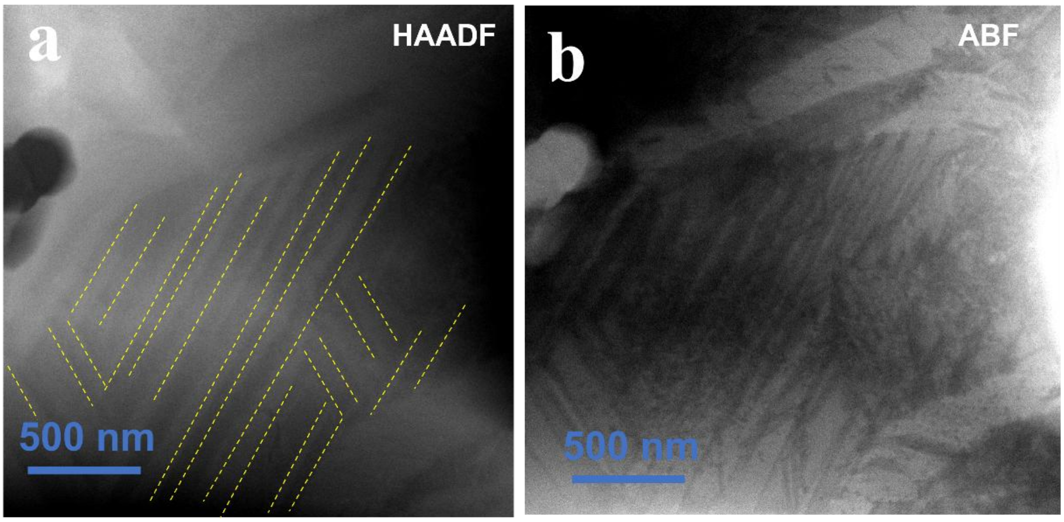

Supplementary Fig. 5. The strip ferroelectric domain structure in $(GeTe)_{0.93}(CuBiS_2)_{0.07}$ sample. (a) A low-magnification HAADF-STEM image. (b) ADF-STEM image of the same area.

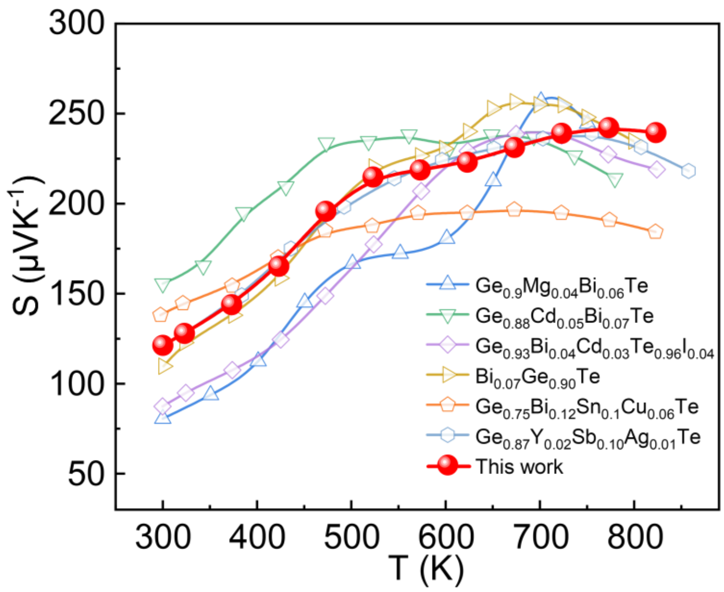

Supplementary Fig. 6. Comparison of the Seebeck coefficient with literature reports.

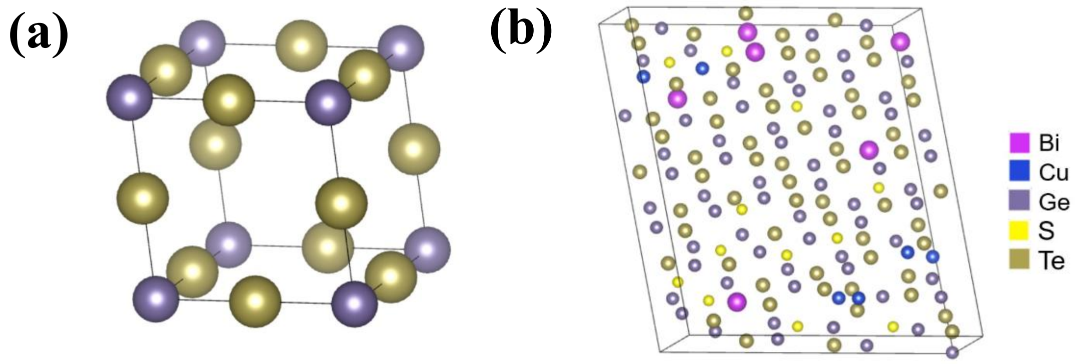

Supplementary Fig. 7. Crystal structures of $(GeTe)_{1-x}(CuBiS_2)_x$ samples. (a) pristine cubic GeTe and (b) $Ge_{63}Cu_6Bi_6Te_{63}S_{12}$.

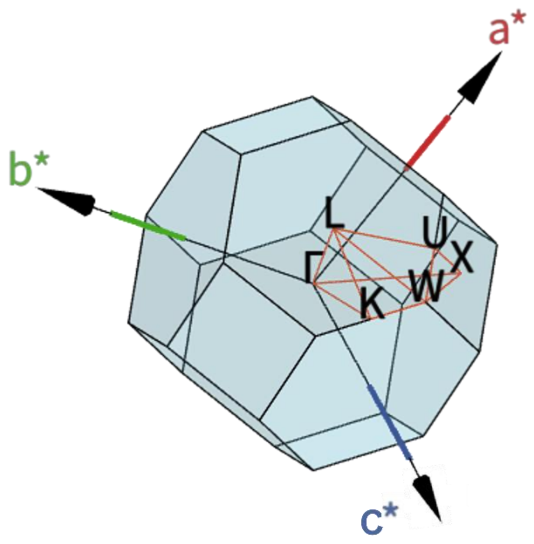

Supplementary Fig. 8. The *k*-point path in the first Brillouin zone for *c*-GeTe.

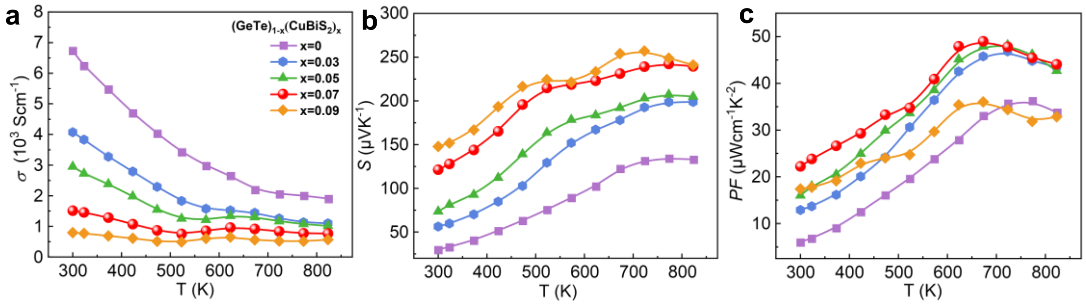

Supplementary Fig. 9. Comparison of the electrical transport properties of the $(GeTe)_{0.91}(CuBiS_2)_{0.09}$ sample with other $CuBiS_2$ alloying samples. (a) Electrical conductivities (σ), (b) Seebeck coefficients (S), (c) power factor (PF).

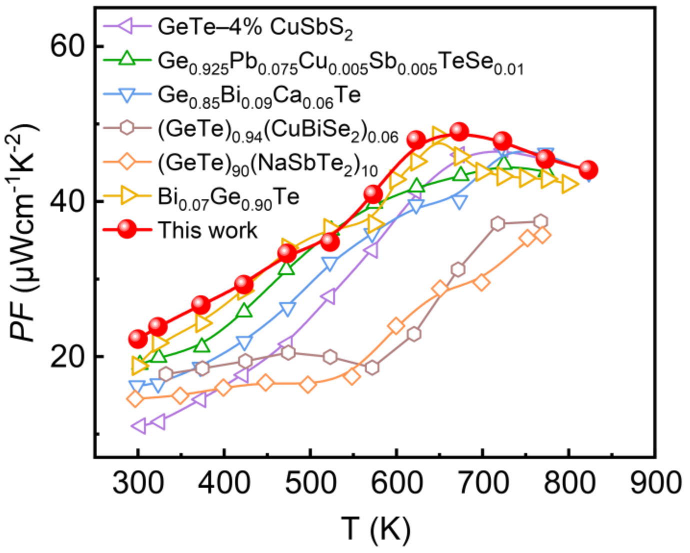

Supplementary Fig. 10. Comparison of the Power factor with literature reports.

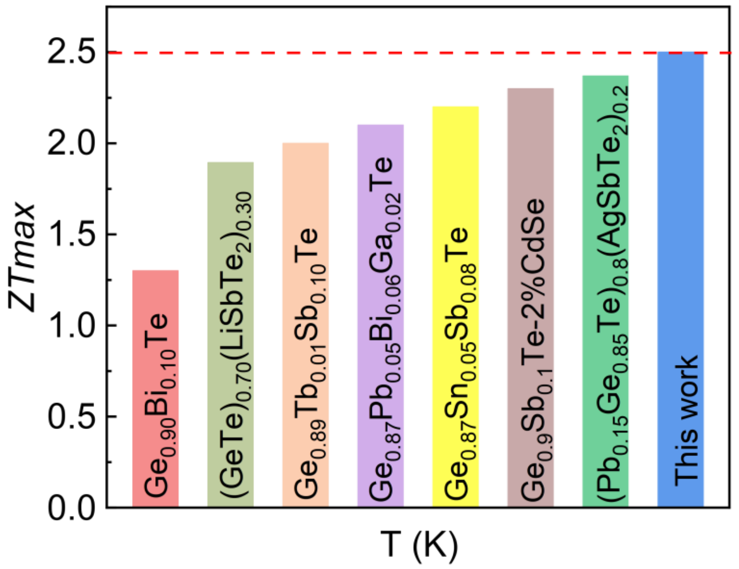

Supplementary Fig. 11. Compared the maximum *ZT* of $(GeTe)_{0.93}(CuBiS_2)_{0.07}$ in this work with other GeTe-based materials.

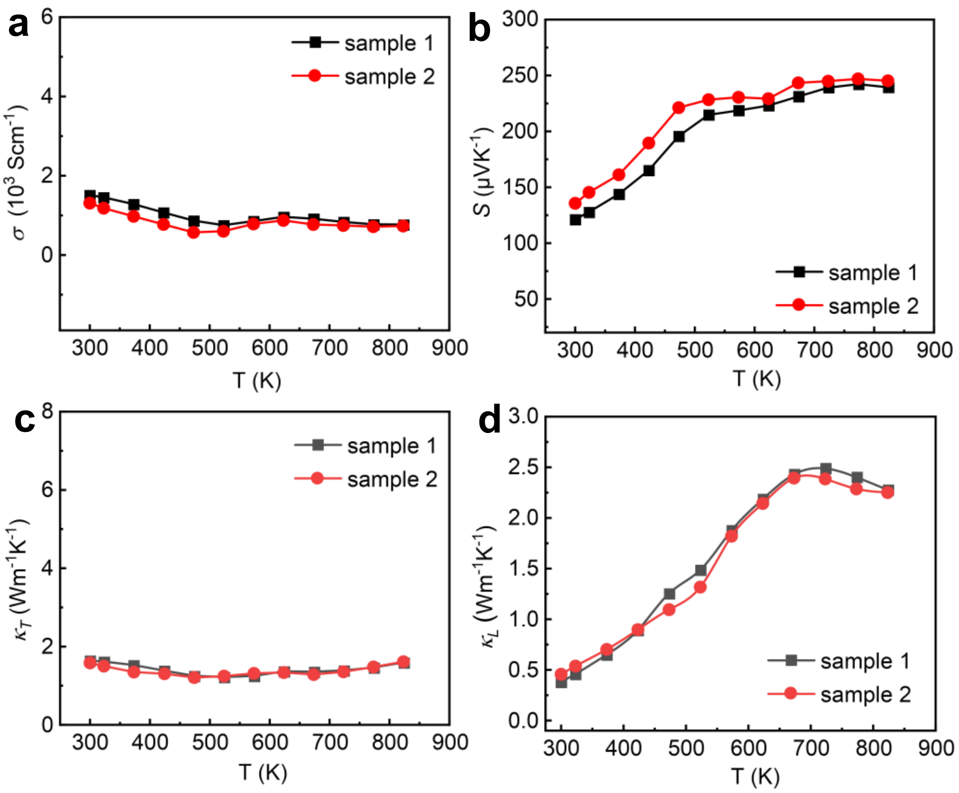

Supplementary Fig. 12. Reproductivity of the high-thermoelectric-performance $(GeTe)_{0.93}(CuBiS_2)_{0.07}$ sample. (a) Electrical conductivity, (b) Seebeck coefficient, (c) Thermal conductivity, (d) *ZT*.

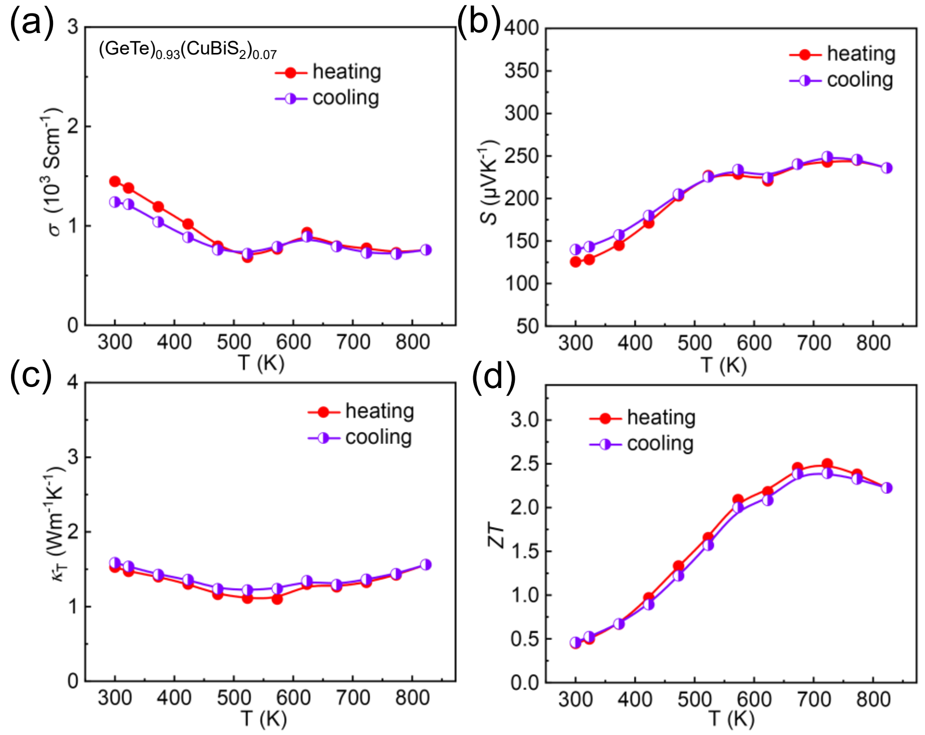

Supplementary Fig. 13. Heating-cooling cycles of $(GeTe)_{0.93}(CuBiS_2)_{0.07}$ sample. (a) Electrical conductivity, (b) Seebeck coefficient, (c) Thermal conductivity, (d) *ZT*.

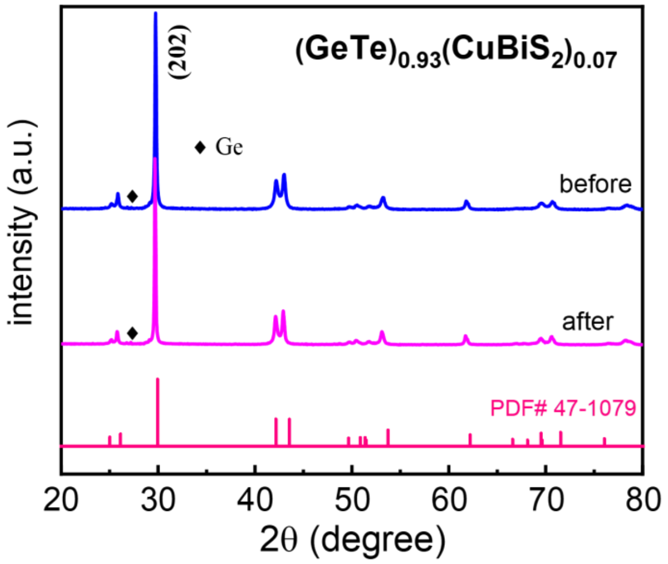

Supplementary Fig. 14. XRD patterns of $(GeTe)_{0.93}(CuBiS_2)_{0.07}$ sample before and after heating-cooling cycling measurement.

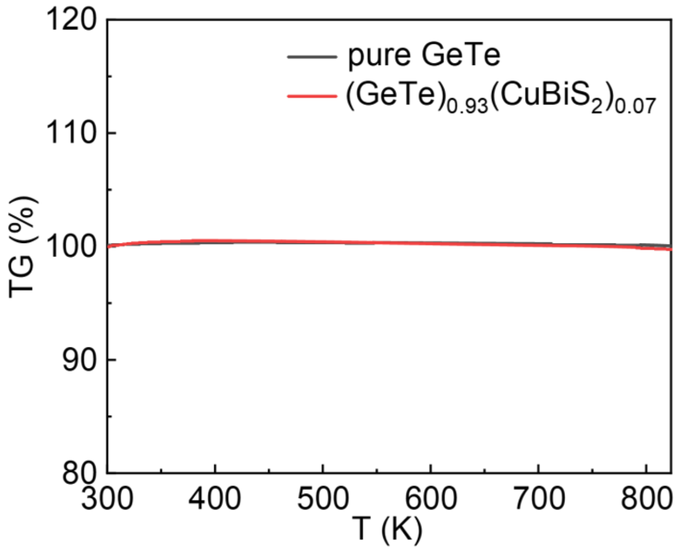

Supplementary Fig. 15. Thermogravimetric analysis for pure GeTe and $(GeTe)_{0.93}(CuBiS_2)_{0.07}$ sample.

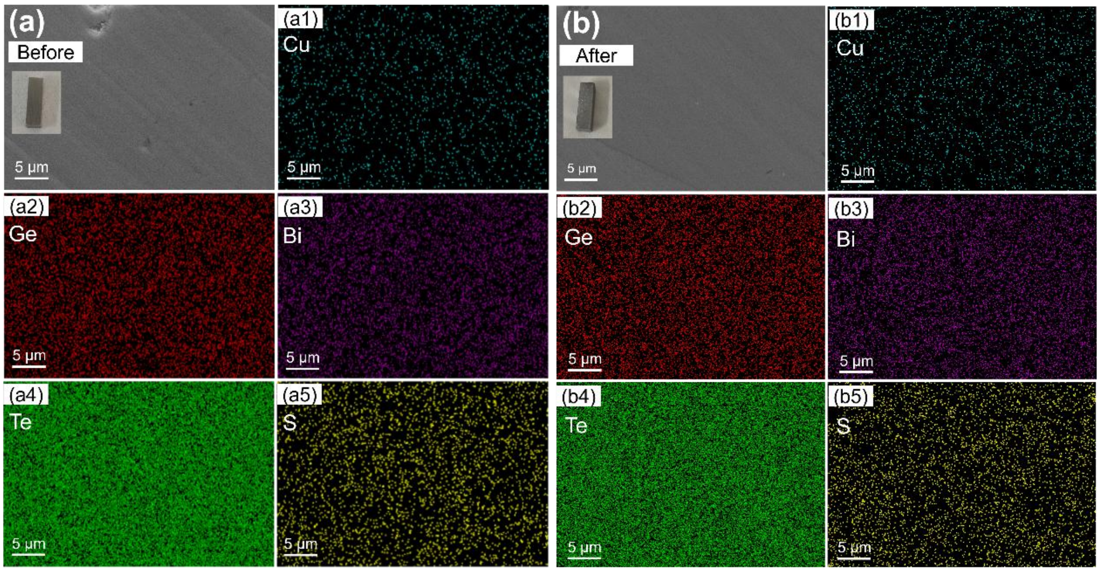

Supplementary Fig. 16. SEM image and corresponding EDS mappings of polished surface of $(GeTe)_{0.93}(CuBiS_2)_{0.07}$ sample. (a) Before, and (b) after heating-cooling cycles measurements. The EDS mappings for Cu, Ge, Bi, Te, and S are displayed in panels (a1-a5) and (b1-b5), respectively.

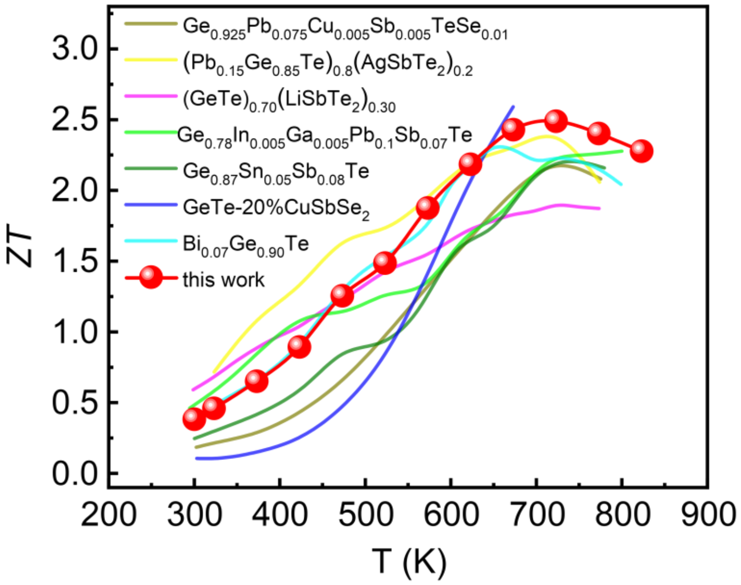

Supplementary Fig. 17. Compared the *ZT* of $(GeTe)_{0.93}(CuBiS_2)_{0.07}$ in this work with other GeTe-based materials in the whole temperature range.

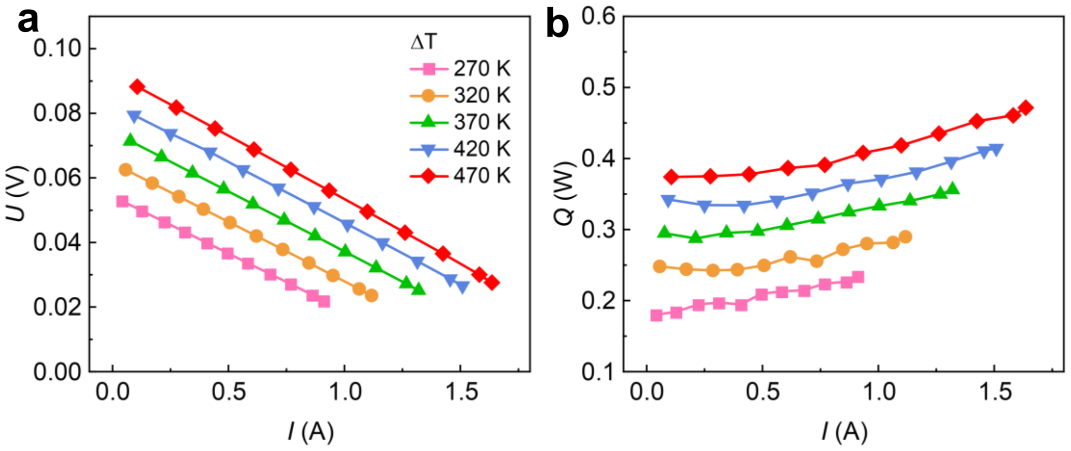

Supplementary Fig. 18. Measurement results of the single leg $(GeTe)_{0.93}(CuBiS_2)_{0.07}$ thermoelectric device. (a) Output voltage (U), (b) input heat flow (Q) as functions of current (I) under various temperature differences (ΔT).

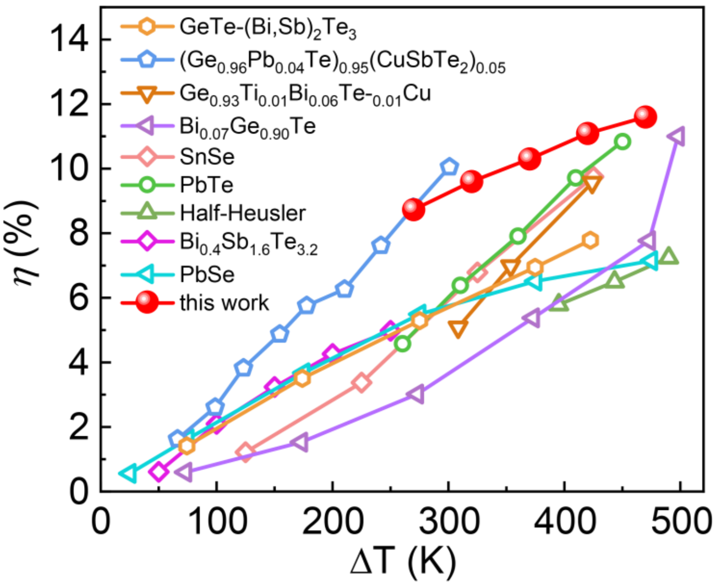

Supplementary Fig. 19. Comparison of measured thermoelectric conversion efficiencies for a $(GeTe)_{0.93}(CuBiS_2)_{0.07}$ single-leg device (this work) with other works among comparable temperature differences ΔT.

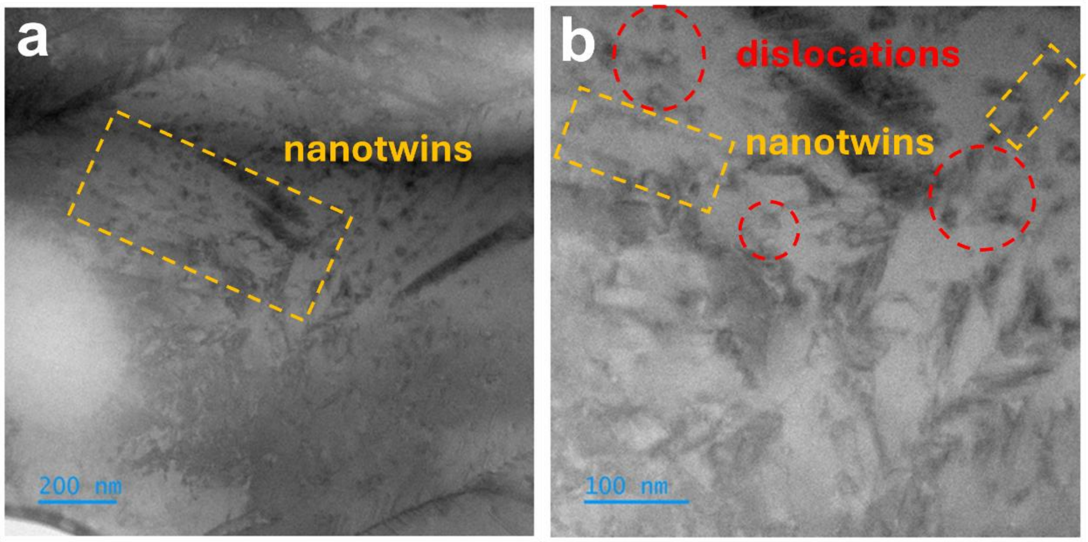

Supplementary Fig. 20. ABF-STEM (Annular Bright-Field Scanning Transmission Electron Microscopy) image (showing strain information) of nanotwins and dislocations. (a) The stripes in the yellow frame show nanotwins. (b) The red circles mark dislocations located among the nanotwins (yellow frames).

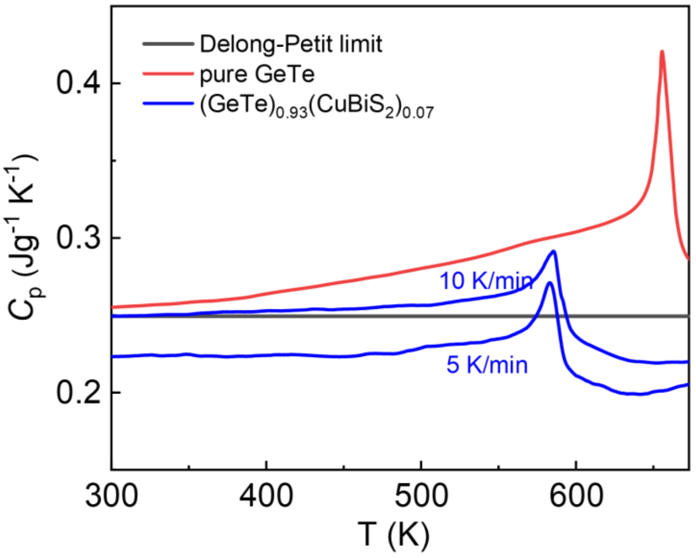

Supplementary Fig. 21. Heat capacity of $(GeTe)_{1-x}(CuBiS_2)_x$ samples by DSC measurements.

The heat capacity (C_p) is measured by NETZSCH DSC 404F3 under different heating rates. C_p decreases after $CuBiS_2$ alloying as compared with pure GeTe. The measured heat capacity of $(GeTe)_{0.93}(CuBiS_2)_{0.07}$ sample is close to Dulong-Petit limit at temperatures not close to that of phase transition. Most literatures reported high performance GeTe thermoelectrics used Dulong-Petit limit for calculating heat capacity.[9, 11, 12] To have a consistent comparison on thermal conductivity and *ZT* of GeTe thermoelectrics, the specific heat capacity (C_p) was calculated used Dulong-Petit limit.

References

1. Kuo, J. J. et al. Grain boundary dominated charge transport in Mg3Sb2-based compounds. *Energy & Environmental Science* **11**, 429-434 (2018).

2. Dou, Y. et al. Lone-pair engineering: Achieving ultralow lattice thermal conductivity and enhanced thermoelectric performance in Al-doped GeTe-based alloys. *Materials Today Physics* **20**, 100497 (2021).

3. Xing, T. et al. Ultralow Lattice Thermal Conductivity and Superhigh Thermoelectric Figure-of-Merit in (Mg, Bi) Co-Doped GeTe. *Adv. Mater.* **33**, e2008773 (2021).

4. Hong, M. et al. Arrays of Planar Vacancies in Superior Thermoelectric Ge1−x−yCdxBiyTe with Band Convergence. *Adv. Energy Mater.* **8**, 1801837 (2018).

5. Gelbstein, Y. et al. Significant lattice thermal conductivity reduction following phase separation of the highly efficient GexPb1–xTe thermoelectric alloys. *physica status solidi (b)* **251**, 1431-1437 (2014).

6. Hong, M. et al. Thermoelectric GeTe with Diverse Degrees of Freedom Having Secured Superhigh Performance. *Adv. Mater.* **31**, e1807071 (2019).

7. Li, S. et al. Band flattening and localized lattice engineering realized high thermoelectric performance in GeTe. *J. Mater. Chem. A* **13**, 32159 (2025).

8. Zheng, Z. et al. Rhombohedral to Cubic Conversion of GeTe via MnTe Alloying Leads to Ultralow Thermal Conductivity, Electronic Band Convergence, and

High Thermoelectric Performance. *J. Am. Chem. Soc.* **140**, 2673-2686 (2018).

9. Hong, M. et al. Strong Phonon-Phonon Interactions Securing Extraordinary Thermoelectric Ge1-xSbxTe with Zn-Alloying-Induced Band Alignment. *J. Am. Chem. Soc.* **141**, 1742-1748 (2019).

10. Sun, Q. et al. Versatile Vanadium Doping Induces High Thermoelectric Performance in GeTe via Band Alignment and Structural Modulation. *Adv. Energy Mater.* **11**, 2100544 (2021).

11. Dong, J. F. et al. Medium-temperature thermoelectric GeTe: vacancy suppression and band structure engineering leading to high performance. *Energy & Environmental Science* **12**, 1396-1403 (2019).

12. Li, J. et al. Low-Symmetry Rhombohedral GeTe Thermoelectrics. *Joule* **2**, 976-987 (2018).
